# *APOL1* Variants, AKI, and Progression to Kidney Failure in People of African Ancestry Living with HIV

**DOI:** 10.1016/j.ekir.2026.106580

**Published:** 2026-05-12

**Authors:** Rachel K.Y. Hung, John W. Booth, Rachel Hilton, Lucy Campbell, Julie Fox, Andrew Ustianowski, Amanda Clarke, Lisa Hamzah, Sarah Schoeman, Kate Bramham, Fiona Burns, Hajra Okhai, Caroline A. Sabin, Cheryl A. Winkler, Frank A. Post, GEN-AFRICA Study Group, John Booth, Anele Waters, James Hand, Chris Clarke, Sarah Murphy, Maurice Murphy, Marion Campbell, Amanda Clarke, Celia Richardson, Alyson Knott, Gemma Weir, Rebecca Cleig, Helena Soviarova, Lisa Barbour, Tanya Adams, Vicky Kennard, Vittorio Trevitt, Rachael Jones, Alexandra Schoolmeester, Serah Duro, Rachel Hilton, Julie Fox, May Rabuya, Lisa Hamzah, Deborah Jordan, Teresa Solano, Hiromi Uzu, Karen Williams, Julianne Lwanga, Linda Ekaette Reid-Amoruso, Hannah Gamlen, Robert J. Stocker, Fiona Ryan, Anele Waters, Karina Mahiouz, Tess Cheetham, Claire Williams, Achyuta Nori, Caroline Thomas, Sivaraj Venkateshwaran, Jessica Doctor, Andrea Berlanga, Frank Post, Beatriz Santana-Suarez, Leigh McQueen, Priya Bhagwandin, Lucy Campbell, Bee Barbini, Emily Wandolo, Tim Appleby, Deborah Jordan, Lois Driver, Sophy Parr, Hongbo Deng, Julie Barber, Andrew Crowe, Chris Taylor, Mary Poulton, Vida Boateng, Marie-Pierre Klein, Caitlin O'Brien, Samuel Ohene-Adomako, Christian Buckingham, Daniel Trotman, Killian Quinn, Kate Flanagan, Verity Sullivan, Holly Middleditch, Itty Samuel, Elizabeth Hamlyn, Candice McDonald, Ana Canoso, Emeka Agbasi, Maria Liskova, Sarah Barber, Amanda Samarawickrama, Zoe Ottaway, Claire Norcross, Amelia Oliveira, Kate Bramham, Sarah Schoeman, Gary Lamont, Ruby Cross, Gaushiya Saiyad, Shadia Ahmed, Rebecca Ashworth, Nicola Window, J. Murira, Khine Phyu, Andrew Ustianowski, Gabriella Lindergard, Jonathan Shaw, Sarah Holland, Claire Fox, Jan Flaherty, Margaret-Anne Bevan, Valerie George, David Chadwick, Marie Branch, Pauline Lambert, Adele Craggs, Sarah Pett, Hinal Lukha, Nina Vora, Marzia Fiorino, Maria Muller Nunez, Deirdre Sally, James E. Burns, Erica Pool, Rebecca Matthews, David Ashley Price, Tara Stothard, Bijal Patel, Ian McVittie, Ciara Kennedy, Uli Shwab, Brendan Payne, Sarah Duncan, Jill Dixon, Mathias Schmid, Adam Evans, Christopher Duncan, Ewan Hunter, Yusri Taha, Natasha Astill, Cheryl Winkler, Elizabeth Binns- Roemer, Victor David, Jonathan Ainsworth, Rachel Vincent, Stephen Kegg, Chloe Saad, Sarah Skinner, Hocine Azzoug, Judith Russell, Tarik Moussaoui, Celia Richardson, Emily Mabonga, Donna Ward, J. Francoise, W. Larbi, Sue Mitchell, A. Manning, V. Russell, Fiona Burns, Mark Harber, Nnenna Ngwu, Jonathan Edwards, Nargis Hemat, Tom Fernandez, Filippo Ferro, Jorge Ferreira, Alice Nightingale, Tasha Oakes-Monger, Darwin Matila, Pedro Nogueira, Victoria Mutagwanya, Catherine Cosgrove, Lisa Hamzah, Catherine Emily Isitt, Helen Webb, Joyce Popoola, Kate Korley, Mark Mencias, Patricia Ribeiro, Rajeshwar Ramkhelawn, Sandra Oliva Lara, Sara Sajijad, Alan Winston, Amber Shaw, Claire Petersen, Kyle Ring, Melanie Rosenvinge, Chloe Saad, Sarah Skinner, Thembi Moyo, Faith Odong, Katherine Gantert, Tina Ibe, Denis Onyango, Caroline Sabin, Teresa Hill, Hajra Okhai

**Affiliations:** 1 King’s College London, London, UK; 2 Barts Health NHS Trust, London, UK; 3 Guy’s and St Thomas’ NHS Foundation Trust, London, UK; 4 Pennine Acute Hospitals NHS Foundation Trust, Manchester, UK; 5 Brighton and Sussex University Hospital NHS Trust, Brighton, UK; 6 Brighton and Sussex Medical School Department of Infectious Disease, Brighton, UK; 7 St George’s Hospital NHS Foundation Trust, London, UK; 8 Leeds Teaching Hospitals NHS Trust, Leeds, UK; 9 King’s College Hospital NHS Foundation Trust, London, UK; 10 University College London, London, UK; 11 Cancer Innovation Laboratory, Center for Cancer Research and the Frederick National Laboratory for Cancer Research, National Cancer Institute, Frederick, Maryland, USA

**Keywords:** apolipoprotein L1, APOL1, chronic kidney disease, Africa, AKI, HIV

## Abstract

**Introduction:**

Acute kidney injury (AKI) is a common complication of HIV infection and associated with progression to kidney failure. We investigated associations between *Apolipoprotein L1* (*APOL1*) variants and AKI, and between AKI, *APOL1* variants and incident kidney failure, in people of African ancestry with HIV.

**Methods:**

We conducted a retrospective analysis of AKI (Kidney Disease: Improving Global Outcomes stages 2/3 and 3) in Genetic Markers of Kidney Disease Progression in People of African Ancestry with HIV (GEN-AFRICA) participants stratified by *APOL1* genotype (2 [G1G1, G1G2, G2G2], 1 [G1G0, G2G0] or 0 [G0G0] variants). Robust Poisson regression estimated AKI prevalence ratios (PR) within 3 months of cohort inception. Poisson regression with generalized estimating equations estimated AKI incidence rate ratios (IRR) during follow-up, adjusting for sex, age, time-updated cluster of differentiation 4 (CD4) count, HIV viral load, and kidney function. Cox proportional hazards models examined associations between AKI, *APOL1* variants, and incident kidney failure.

**Results:**

Among 2701 participants (43% male; mean age 38.7 years; 12.7% with 2 APOL1 variants), 165 individuals experienced 183 AKI episodes. Participants with 2 APOL1 variants were more likely to experience AKI at cohort inception (adjusted PR 6.9, 95% CI: 3.5–13.8, *P* < 0.001), had more severe AKI, and poorer recovery of kidney function post-AKI compared with those with 0 variants, but not during follow up. AKI was associated with incident kidney failure and this association varied by APOL1 status (*P*_interaction_ < 0.001).

**Conclusion:**

Among people with HIV, 2 APOL1 variants were associated with increased risk of AKI, especially severe AKI at cohort inception, and poorer recovery of post-AKI. AKI identified individuals at increased risk of progression to kidney failure.

## Graphical abstract

**Figure:**
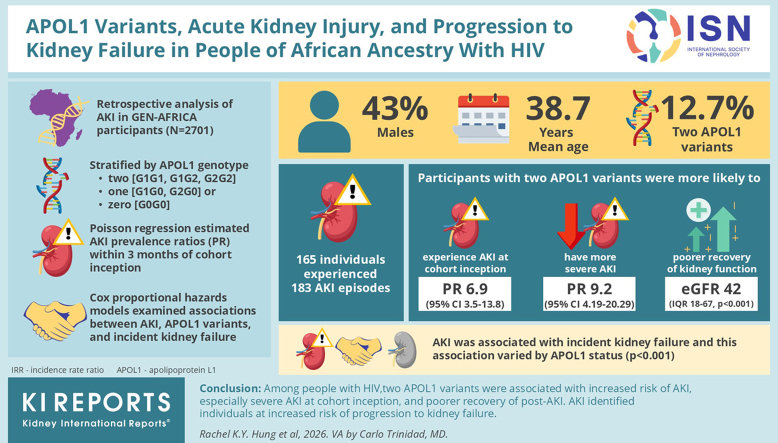

AKI is a common and clinically significant complication of HIV infection.^1^ AKI often occurs in the context of severe illness, severe immunosuppression, and high levels of HIV replication.^1^^,^^2^ AKI is recognized as an independent risk factor for chronic kidney disease (CKD), kidney failure and death,^3^^,^^4^ with growing evidence that a substantial proportion of AKI survivors do not fully recover kidney function.^5^ Although expanded access to antiretroviral therapy (ART) has attenuated some of the traditional risk factors for AKI such as opportunistic infections and advanced immunodeficiency, people living with HIV continue to experience a disproportionately higher risk of both AKI and subsequent CKD. Notably, this increased risk persists even among those with sustained virological suppression.^6^

*Apolipoprotein L1 (APOL1*) genetic variants have been implicated in the increased risk of CKD and kidney failure in people of African ancestry.^7^, ^8^, ^9^ People with HIV who have *APOL1* high-risk genotypes (2 variants) experienced faster decline of estimated glomerular filtration rate (eGFR),^10^^,^^11^ and this decline is accelerated in those with HIV viraemia.^12^^,^^13^ Although the association between *APOL1* high-risk variants and proteinuric CKD is well established, their relationship with AKI remains less clear. During the COVID-19 pandemic*,* people with *APOL1* high-risk genotypes with COVID-19 were found to be at higher risk of developing AKI, more severe AKI, and death.^14^^,^^15^ However, the contribution of *APOL1* variants to AKI outside the context of COVID-19 infection, particularly among people living with HIV and over the long term, remains poorly described. Existing studies in non-HIV populations^14^^,^^16^ have focused on acute episodes or short-term outcomes, leaving a gap in our understanding of *APOL1*-associated risk of AKI in people with HIV, both at HIV diagnosis and during follow up on ART.

In this study, we used longitudinal data from the GEN AFRICA cohort to study the association of *APOL1* variants and AKI in people with HIV. We aimed to determine the incidence of AKI, severity of AKI, and recovery from AKI by *APOL1* status, and whether AKI was associated with progression to kidney failure.

## Methods

We conducted a retrospective analysis of participants in the GEN-AFRICA study (NCT05685810). The GEN-AFRICA study was open to individuals of black ethnicities aged ≥ 18 years living with HIV; they were enrolled across 15 HIV clinics and 3 dialysis/kidney transplantation centers in England between May 2018 and February 2020. As described previously,^8^ demographic and clinical information was obtained during a single study visit via questionnaires corroborated through review of the medical records. Serial laboratory data including HIV viral loads, CD4 cell counts, urine protein creatinine ratio (uPCR) and creatinine measurements from the time of initiation of HIV care to December 31, 2024 were obtained through linkage with electronic health records.

The exposure of interest was *APOL1* status which was categorized according to the number of *APOL1* variants (2 [G1G1, G1G2, G2G2], 1 [G1G0, G2G0], or 0 [G0G0]). The primary outcome was AKI stage 2/3, based on KDIGO serum creatinine criteria (AKI stage 2: increase in serum creatinine of 2.0–2.9 times over baseline, AKI stage 3: increase of 3.0 times over baseline value, or a rise in creatinine to at least 354 μmol/l with a > 1.5-fold increment over the most recent creatinine [previous 3-month interval; to mitigate CKD progression being mislabeled as AKI). We assessed kidney function pre- and post-AKI using eGFR, calculated with the CKD-Epidemiology Collaboration (2021) equation.^17^ The secondary outcome was kidney failure defined by the presence of eGFR < 15 ml/min per 1.73 m^2^ for at least 3 months.

### Statistical Analysis

Cohort inception (baseline) was defined by the date of the first available serum creatinine measurement following HIV diagnosis. At cohort inception, participants were categorized as having a new HIV diagnosis, re-engaged with HIV services (HIV viraemia (≥ 200 copies/mL) with a previous HIV diagnosis and/or ART start date), or having transferred their HIV care to a participating center (suppressed HIV (< 200 copies/ml), with a previous HIV diagnosis and/or ART start date). Baseline characteristics of the study participants, stratified by *APOL1* and AKI status, were described and compared using the chi square test for categorical variables and Kruskal–Wallis tests or analysis of variance for continuous variables, as appropriate. For descriptive purposes, HIV viral load values were transformed to the log_10_ scale when reporting median viral load (e.g., 50 copies/ml ≈ log_10_ 1.7; 100,000 copies/ml ≈ log_10_ 5).

As AKI in people with HIV is considerably more common around the time of HIV diagnosis,^3^ we analyzed AKI episodes that occurred at cohort inception (based on creatinine measurements up to 3 months from cohort inception), and episodes that occurred during clinical follow up (from 3 months onwards) separately. AKI episodes within 3 months of cohort inception were analyzed cross-sectionally using robust Poisson regression to describe associations between *APOL1* variant status and AKI, with Wald tests to assess the strength of association at each level. Multivariable models were adjusted for age, sex, CD4 cell count (< 50, 50–199, 200–349 and ≥ 350 cells/mm^3^) and HIV viral load. Because of low AKI event numbers in several HIV viral load strata, categories were merged to ensure robust estimation of effect estimates and model stability. HIV viral load was categorized to < 5000, 5000–100,000 and ≥ 100,000 copies/ml in the first 3 months and as undetectable versus detectable (< 50 and ≥ 50 copies/ml) thereafter.

Kidney function at 3 months postcohort inception was described, when acute illness and AKI episodes had largely resolved and participants had had an opportunity to access ART (as per prevailing national guidelines). AKI episodes during subsequent follow-up were analyzed from this timepoint forward in participants without kidney failure (i.e., eGFR > 15ml/min per 1.73 m^2^), by comparing creatinine measurements to those obtained 3 months previously; follow-up time was calculated to the last visit or the onset of kidney failure, whichever occurred first. Each person’s follow-up time was split into a series of consecutive 3-month intervals. Each 3-month interval was assigned the most recent creatinine (highest creatinine if > 1 measurement was available), CD4 cell count (< 50, 50–199, 200–349, ≥ 350 cells/mm^3^), and HIV viral load (< 50 and ≥ 50 copies/ml); uPCR was categorized as < 15, 15–49, 50–99, ≥ 100 mg/mmol and eGFR was calculated from the assigned creatinine value and categorized as < 60, 60–89, ≥ 90 ml/min per 1.73 m^2^). Crude and adjusted AKI IRR were calculated using generalized estimating equation Poisson models with robust estimation to allow for multiple episodes of AKI within an individual. AKI was considered to have resolved after 3 months, after which participants became again at risk of further episodes of AKI unless kidney failure had ensued. All models were adjusted for age and sex, and for time-updated CD4 cell count, HIV viral load, and recent eGFR and uPCR; eGFR and uPCR (measurements from the preceding 3 months). Sensitivity analyses were performed to describe the associations with AKI Stage 3 only. Missing urine PCR values were considered likely missing at random, as the decision to obtain uPCR in clinical practice is usually based on observed characteristics—such as abnormal creatinine, comorbidities, or suspected kidney disease—rather than by the unobserved uPCR value itself. A degree of missing not at random cannot be excluded, given that the underlying degree of proteinuria may also influence whether testing is performed. Owing to this substantial missingness, we repeated the models without this variable to confirm that the results were not dependent on its inclusion.

To explore the relationship between *APOL1* status, AKI and kidney failure, we graphically displayed the cumulative incidence of kidney failure by *APOL1* status using Kaplan-Meier methods, and used Cox proportional hazards modelling to estimate the crude and adjusted hazard ratios for developing kidney failure from 3 months after cohort inception onwards; models were adjusted for age and sex, *APOL1* status (those with 0 variants were the referent group), and time-updated AKI status (ever vs. never), CD4 cell count and HIV viral load. The proportional hazards assumption was assessed by visual inspection. All statistical analyses were done using R Statistical Software (v4.4.0, R Foundation, Vienna, Austria).

## Results

A total of 3027 individuals were enrolled in the GEN-AFRICA study, with successful *APOL1* genotyping in 2864 (94.6%). Among these 2701 participants with known *APOL1* status and available creatinine measurements before enrolment were included in analyses of AKI at cohort inception, whereas 2635 participants were included in analyses of AKI during follow-up (Supplementary Figure S1). Participants had a mean age of 38.7 (SD 10.8) years at cohort inception, 43% were male, and 343 (12.7%), 1033 (38.2%) and 1325 (49.1%) had 2, 1, or 0 *APOL1* variants, respectively. The prevalence of 2 *APOL1* variants was highest among individuals of West African ancestry. The median CD4 cell count was 314 (IQR 131–516) cells/mm^3^ and the median HIV viral load 3.6 (1.7– 4.8) log copies/ml. Participants with 2 *APOL1* variants had a lower median eGFR at cohort inception as compared with those with 1 or 0 variants (81 vs. 98 vs. 95 ml/min per 1.73 m^2^, respectively) (Table 1). Characteristics at cohort inception by AKI status during follow up are additionally shown in Supplementary Table S1.

**Table 1:** Baseline characteristics of participants by *APOL1* genotype at cohort inception

Participant characteristics		Overall	0 *APOL1* variant	1 *APOL1* variant	2 *APOL1* variants	
		(*N* = 2701)	(*n* = 1325)	(*n* = 1033)	(*n* = 343)	
Demographics						
Age (yrs)	Mean (SD)	38.7 (10.8)	38.3 (10.7)	39.0 (10.9)	39.5 (10.5)	0.1
Ancestry						< 0.001
East Africa	*n* (%)	537 (19.9)	423 (31.9)	104 (10.1)	10 (2.9)	
South Africa	*n* (%)	671 (24.8)	374 (28.2)	252 (24.4)	45 (13.1)	
Central Africa	*n* (%)	149 (5.5)	85 (6.4)	54 (5.2)	10 (2.9)	
West Africa	*n* (%)	861 (31.9)	240 (18.1)	405 (39.2)	216 (63.0)	
Caribbean	*n* (%)	400 (14.8)	165 (12.5)	182 (17.6)	53 (15.5)	
Other	*n* (%)	76 (2.8)	35 (2.6)	32 (3.1)	9 (2.6)	
Sex (male)	*n* (%)	1168 (43.2)	548 (41.4)	459 (44.4)	161 (46.9)	0.2
HIV status at cohort inception						0.1
New HIV diagnosis	*n* (%)	1467 (54.3)	690 (52.1)	576 (55.8)	201 (58.6)	
Reengagement with care	*n* (%)	233 (8.6)	128 (9.7)	82 (7.9)	23 (6.7)	
Transfer into care	*n* (%)	999 (37.0)	505 (38.1)	375 (36.3)	119 (34.7)	
CD4 cell count (cells/mm^3^)^a^	Median (IQR)	314 (131–516)	309 (119–510)	273 (85–463)	307 (122–509)	0.03
HIV viral load (log copies/ml)^a^	Median (IQR)	3.6 (1.7–4.8)	3.8 (1.7–4.9)	3.8 (1.7–4.9)	3.7 (1.7–4.8)	0.2
Kidney function at cohort inception^a^						
eGFR (ml/min per 1.73 m^2^)	Median (IQR)	95 (78–110)	98 (81–112)	95 (79–109)	81 (45–98)	< 0.001
eGFR Stage 1 (> 90)	*n* (%)	1546 (57.2)	817 (61.7)	597 (57.8)	132 (38.5)	
eGFR Stage 2 (60–90)	*n* (%)	870 (32.2)	415 (31.3)	349 (33.8)	106 (30.9)	
eGFR Stage 3 (30–59)	*n* (%)	169 (6.3)	63 (4.8)	69 (6.7)	37 (10.8)	
eGFR Stage 4 (15–29)	*n* (%)	33 (1.2)	16 (1.2)	4 (0.4)	13 (3.8)	
eGFR Stage 5 (< 15)	*n* (%)	83 (3.1)	14 (1.1)	14 (1.4)	55 (16.0)	
Urine PCR (mg/mmol)^b^	Median (IQR)	11 (7–23)	11 (7–20)	10 (7–18)	23 (8–172)	< 0.001
uPCR < 15	*n* (%)	458 (17.0)	216 (16.3)	193 (18.7)	49 (14.3)	
uPCR 15–49	*n* (%)	161 (6.0)	79 (6.0)	60 (5.8)	22 (6.4)	
uPCR 50–99	*n* (%)	36 (1.3)	18 (1.4)	12 (1.2)	6 (1.7)	
uPCR > 100	*n* (%)	72 (2.7)	18 (1.4)	17 (1.6)	37 (10.8)	
uPCR measurements unavailable	*n* (%)	1974 (73.1)	994 (75.0)	751 (72.7)	229 (66.8)	

Ancestry information was unavailable for n = 7, HIV status for n = 2, CD4 cell count for n = 1, HIV RNA for n = 1.

a First available reading after cohort inception.

b Urine PCR within 3 months of cohort entry unavailable for 1974 (73.1%).

### AKI at Cohort Inception

Fifty-nine participants experienced AKI at cohort inception (26, 17, and 16 in those with 2, 1, and 0 *APOL1* variants, respectively), mostly in a setting of severe immunodeficiency (median CD4 cell count 61 cells/mm^3^) and high levels of HIV replication (median HIV viral load 5.5 log copies/ml). When compared with those with 0 variants, participants with 2 *APOL1* variants experienced more severe AKI (median peak creatinine 678 vs. 315 μmol/l), with more severe proteinuria (median uPCR 529 vs. 125 mg/mmol) and poorer recovery post-AKI (median eGFR 42 vs. 77 ml/min per 1.73 m^2^) (Table 2). In those with 2 *APOL1* variants, proteinuria generally persisted post-AKI (median uPCR 502 vs. 7 mg/mmol). Participants with 1 *APOL1* variant experienced less severe AKI (median peak creatinine 198 μmol/l) and better recovery (median eGFR 89 ml/min per 1.73 m^2^) as compared with those with 0 *APOL1* variants. The prevalence of stage 2/3 AKI at cohort inception was 7.6%, 1.6%, and 1.2 % among those with 2, 1, and 0 *APOL1* variants (*P* < 0.001). Having 2 *APOL1* variants was associated with AKI in univariable analysis (PR 6.3, 95% CI: 3.4–11.6, *P* < 0.001) and remained strongly associated with AKI in multivariable analysis after adjustment for age, sex, HIV viral load and CD4 cell count (adjusted PR 6.9, 95% CI: 3.5–13.8, *P* < 0.001). When the analyses were restricted to AKI Stage 3, stronger associations were observed (PR 7.4 [3.7–14.7]; adjusted PR 9.2 [4.2–20.3] *P* < 0.001) (Table 3; full models are shown in Supplementary Table S2 and S3). A single *APOL1* variant was not associated with stage 2/3 or stage 3 AKI.

**Table 2:** Clinical characteristics of the study participants around the time of developing AKI

All AKI episodes		All	0 *APOL1* variant	1 *APOL1* variant	2 *APOL1* variants	*P*-value
		*N* = 183	*n* = 73	*n* = 66	*n* = 44	
Age (SD)	Mean (SD)	45.3 (12.0)	46.6 (12.2)	46.3 (12.5)	41.6 (10.5)	0.01
Sex (Male)	*n* (%)	95 (51.9)	35 (47.9)	40 (60.6)	20 (45.5)	0.4
Severity of AKI (KDIGO stage)						
Stage 2	*n* (%)	80 (44)	37 (51)	33 (50)	10 (23)	< 0.001
Stage 3	*n* (%)	103 (56)	36 (49)	33 (50)	34 (77)	< 0.001
AKI episodes at inception^a^	N	*N* = 59	*n* = 16	*n* = 17	*n* = 26	
CD4 cell count (cells/mm^3^)	Median (IQR)	61 (18–189)	74 (47–229)	58 (18–193)	31 (14–178)	0.3
HIV viral load (log copies/ml)	Median (IQR)	5.5 (3.9–5.9)	5.2 (3.1–5.5)	5.3 (3.2–6.1)	5.7 (5.5–5.8)	0.2
Pre AKI						
Creatinine	Median (IQR)	314 (157–618)	233 (136–384)	165 (150–246)	497 (318–797)	< 0.001
eGFR	Median (IQR)	17 (9–41)	22 (16–24)	39 (32–46)	11 (5–14)	< 0.001
At AKI						
Peak creatinine	Median (IQR)	375 (195–713)	315 (189–449)	198 (155–351)	678 (353–840)	< 0.001
Nadir eGFR	Median (IQR)	14 (8–35)	18 (13–34)	36 (18–41)	8 (5–14)	< 0.001
uPCR (*n* = 25 [6, 6, 13])	Median (IQR)	289 (69–954)	125 (74–166)	78 (24–173)	529 (300–1050)	0.05
Post AKI						
Creatinine	Median (IQR)	112 (83–198)	106 (84–138)	79 (66–108)	172 (105–297)	0.01
eGFR	Median (IQR)	67 (30–93)	77 (42–102)	89 (64–114)	42 (18–67)	< 0.001
uPCR (*n* = 14 [4, 2, 8])	Median (IQR)	176 (17–536)	7 (4–12)	28 (23–34)	502 (316–596)	0.007
AKI episodes during follow up^b^	N	124^c^	57	49	18	
CD4 cell count (cells/mm^3^)	Median (IQR)	327 (178–554)	378 (197–636)	256 (82–421)	275 (204–446)	0.10
HIV viral (log copies/ml)	Median (IQR)	1.7 (1.6–3.9)	1.7 (1.6–2.8)	1.7 (1.6–3.9)	1.8 (1.5–5.3)	0.70
Pre AKI						
Creatinine	Median (IQR)	88 (67–114)	81 (65–110)	92 (75–107)	119 (70–185)	0.20
eGFR	Median (IQR)	83 (60–110)	84 (67–106)	87 (68–112)	58 (34–105)	0.30
uPCR (*n* = 30 [14, 10, 6])	Median (IQR)	20 (9–191)	27 (15–59)	12 (9–24)	241 (30–438)	0.40
At AKI						
Peak creatinine	Median (IQR)	245 (179–369)	251 (171–359)	234 (183–319)	365 (187–693)	0.20
Nadir eGFR	Median (IQR)	24 (14–37)	23 (16–36)	27 (18–36)	13 (8–31)	0.10
uPCR (n=44 [18, 16, 10])	Median (IQR)	50 (13–178)	37 (14–151)	21 (12–81)	310 (63–741)	0.10
Post AKI						
Creatinine (*n* = 118 [53, 48, 17])	Median (IQR)	100 (78–161)	91 (73–116)	102 (80–153)	250 (108–532)	< 0.001
eGFR (*n* = 118 [53,48, 17])	Median (IQR)	74 (41–92)	82 (54–99)	70 (45–94)	23 (10–70)	< 0.001
uPCR (*n* = 40 [14, 16, 10])	Median (IQR)	43 (17–151)	19 (15–107)	42 (20–70)	597 (154–1270)	0.004

AKI, acute kidney injury; eGFR, estimated glomerular filtration rate (CKD-EPI-2021).

eGFR is expressed as ml/min per 1.73 m^2^; creatinine is expressed in μmol/l and urine protein/creatinine ratio (uPCR) in mg/mmol. For uPCR measurements, the total number of observations is shown with numbers in brackets indicating counts in the 0-, 1-, and 2-*APOL1* risk variant groups, respectively. Pre-AKI at inception and nadir eGFR values were calculated using first available and peak serum creatinine measurements respectively; as kidney function was not in steady state at this time, these values and uPCR should be viewed as descriptive contextual markers only and not as estimates of glomerular filtration.

NB: HIV viral load is expressed as log copies/ml (a viral load of < 50 equates to log 1.7).

a Within 3 months of cohort inception.

b More than 3 months from cohort inception.

c Data available for *n* = 118.

**Table 3:** Associations between *APOL1* variant status and the development of AKI

At cohort inception	Univariable analysis			Multivariable analysis		
	PR	95% CI	*P*-value	PR	95% CI	*P*-value
Stage 2/3 AKI						
1 *APOL1* variant	1.36	069 – 2.68	0.37	1.25	0.55 – 2.82	0.59
2 *APOL1* variants	6.28	3.41 – 11.57	< 0.001	6.91	3.46 – 13.81	< 0.001
Stage 3 AKI						
1 *APOL1* variant	1.49	0.69 – 3.22	0.30	1.29	0.50 – 3.35	0.60
2 *APOL1* variants	7.40	3.72 – 14.73	< 0.001	9.22	4.19 – 20.29	< 0.001


During follow up	IRR	95% CI	*P* value	IRR	95% CI	*P*-value
Stage 2/3 AKI						
1 *APOL1* variant	1.21	0.83 – 1.77	0.33	0.82	0.37 – 1.84	0.63
2 *APOL1* variants	1.67	0.99 – 2.84	0.06	1.67	0.61 – 4.60	0.32
Stage 3 AKI						
1 *APOL1* variant	1.13	0.63 –2.03	0.69	0.68	0.19 – 2.45	0.55
2 *APOL1* variants	2.54	1.28 – 5.06	0.008	1.33	0.30 – 5.83	0.71

AKI, acute kidney injury; IRR, incidence rate ratio; PR, prevalence ratio.

Robust Posson regression was used to estimate the prevalence ratio of AKI for participants with 2 or 1 *APOL1* variants at (within 3 months of) cohort inception, and Poisson regression to estimate the incidence rate ratio of AKI during follow up (more than 3 months from cohort inception).

Participants with 0 *APOL1* variant are the referent group in these analyses. The multivariable models were adjusted for age, sex, CD4 cell count, HIV viral load (< 5000, 5000–100,000 and ≥ 100,000 copies/ml; AKI at cohort inception), and for age, sex and time-updated CD4 cell count, HIV viral load (< 50 and ≥ 50 copies/ml), eGFR (≥ 90, 60–89, < 60 ml/min per 1.73 m^2^) and uPCR (< 15, 15–59, 50–99 and ≥ 100 mg/mmol; AKI during follow up).

### Kidney Disease Status at 3 Months Postcohort Inception

After the acute presentation, the median eGFR at 3 months was 84, 95, and 99 ml/min per 1.73 m^2^ for participants with 2, 1, and 0 *APOL1* variants, respectively. CKD was present in 26.8% of individuals with 2 variants, as compared with 5.7% and 4.9% among those with 1 and 0 variants. Furthermore, 10.3% of participants with 2 *APOL1* variants had eGFR <15 ml/min per 1.73 m^2^, compared with only 1.0% and 2.2% of those with 1 and 0 variant(Table 4).

**Table 4:** Kidney function at 3 months postcohort inception

		All	0 *APOL1* variant	1 *APOL1* variant	2 *APOL1* variants	*P*-value
		(*N* = 2685)	(n = 1314)	(n = 1031)	(n = 340)	
eGFR (ml/min per 1.73 m^2^)	Median (IQR)	96 (80–111)	99 (83–113)	95 (81–110)	84 (57–101)	< 0.001
eGFR stage						
Stage 1 (> 90)	*n* (%)	1594 (59.4)	848 (64.5)	610 (59.2)	136 (40.0)	< 0.001
Stage 2 (60–90)	*n* (%)	877 (32.7)	401 (30.5)	363 (35.2)	113 (33.2)	
Stage 3 (30–59)	*n* (%)	123 (4.6)	44 (3.3)	40 (3.9)	39 (11.5)	
Stage 4 (15–29)	*n* (%)	32 (1.2)	8 (0.6)	7 (0.7)	17 (5.0)	
Stage 5 (< 15)	*n* (%)	59 (2.2)^a^	13 (1.0)	11 (1.1)	35 (10.3)	
uPCR (mg/mmol)	Median (IQR)	9.3 (6.6–16.7)	9.1 (6.5–15.1)	9.1 (6.4–16.0)	11.0 (7.1–62.0)	< 0.001
uPCR < 15	*n* (%)	947 (35.3)	457 (34.8)	374 (36.3)	116 (34.1)	0.01
uPCR 15–49	*n* (%)	244 (9.1)	113 (8.6)	105 (10.2)	26 (7.6)	
uPCR 50–99	*n* (%)	54 (2.0)	23 (1.8)	19 (1.8)	12 (3.5)	
uPCR > 100	*n* (%)	78 (2.9)	20 (1.5)	19 (1.8)	39 (11.5)	
uPCR measurements unavailable	*n* (%)	1362 (50.7)	701 (53.3)	514 (49.9)	147 (43.2)	
HIV viral load (log copies/ml)	Median (IQR)	1.9 (1.7–3.6)	1.9 (1.7–3.7)	1.8 (1.7–3.7)	1.9 (1.7–3.4)	0.50
CD4 cell count (cells/mm^3^)	Median (IQR)	341 (179–536)	356 (180–543)	350 (178–534)	295 (170–517)	0.06

uPCR, urine protein creatinine ratio.

a Includes n = 50 with kidney failure and n = 9 with eGFR <15 and subsequent measurements ≥15 ml/min per 1.73 m^2^.

### AKI During Clinical Follow Up (From 3 Months Postcohort Inception)

Among 2635 participants without kidney failure (340, 1031, and 1314 with 2, 1, and 0 *APOL1* variants, respectively), the median follow-up was 12 (IQR 6.5–16), 13 (IQR 8–17), and 13 (IQR 8.4–18) years for those with 2, 1, and 0 *APOL1* variants, respectively. Over this period, participants contributed 157,586 creatinine measurements, with a median of 38 per person (IQR 19–68). At the start of follow up, the median HIV viral load was 1.9 log [80] copies/ml (IQR 1.7–3.6) and the median CD4 count 341 cells/mm^3^ (IQR 179–536), similar across all *APOL1* genotype groups. During follow up, 113 participants experienced 124 episodes of AKI (18 episodes in those with 2 *APOL1* variants and 49 and 57 in those with 1 and 0 variants respectively; 103 participants experienced 1 episode, 9 had 2 episodes, and 1 had 3 episodes of AKI).

Participants with 2, 1, and 0 *APOL1* variants had similar pre-AKI and AKI characteristics (Table 2), albeit those with 2 *APOL1* variants had somewhat lower pre-AKI eGFR (58 vs. 92 and 81 ml/min per 1.73 m^2^) and somewhat higher peak creatinine measurements (365 vs. 234 and 251 mmol/l). Post-AKI kidney function differed by *APOL1* status, with poor recovery and severe proteinuria in those with 2 variants, incomplete recovery and mild proteinuria in those with 1 variant, and almost complete recovery and no proteinuria in those with 0 *APOL1* variants (Table 2).

The overall incidence of AKI during follow up was 0.62 (95%CI: 0.52–0.74) per 100 person years, with the lowest in those with 0 variants (0.56 [0.42–0.72] as compared with those with 1 (0.65 [0.48–0.86]) and 2 *APOL1* variants (0.85 [0.51–1.35]). Having 2 or 1 *APOL1* variants was not significantly associated with stage 2/3 AKI in univariable analysis, and in multivariable models adjusting for age, sex, and time-updated CD4 cell count, HIV viral load, eGFR, and uPCR (adjusted IRR for 2 variants: 1.67 [0.61–4.60], *P* = 0.32; for 1 variant: 0.82 [0.37–1.84], *P* = 0.63). Although *APOL1* high-risk status (2 variants) was associated with an increased incidence of severe (stage 3) AKI in univariable analysis (IRR 2.54 [1.28–5.06], *P* = 0.008), this association was no longer significant after adjustment (IRR 1.33 [0.30–5.83], *P* = 0.71), and no association was observed for a single *APOL1* variant (Table 3 and Supplementary Tables S4 and S5); similar effect estimates were observed in models excluding uPCR (Supplementary Table S6).

### Association Between AKI and Progression to Kidney Failure

Of the 2635 participants without kidney failure at the start of follow up, 69 progressed to kidney failure (29 [8.5%], 11 [1.1%], and 29 [2.2%] of those with 2, 1 and 0 *APOL1* variants, respectively). At 15 years post-inception, the cumulative incidence of kidney failure was 12% in participants with 2 *APOL1* variants as compared with 2% and 3% in those with 1 and 0 variants, respectively (Supplementary Figure S2). Of the 69 individuals who ultimately developed kidney failure, 8 of 29 (28%), 3 of 11 (27%), and 8 of 29 (28%) with 2, 1 and 0 *APOL1* variants experienced AKI before the onset of kidney failure. By contrast, the occurrence of AKI was much lower among the 2566 individuals who did not progress to kidney failure— 10 of 279 (3.6%) with 2 variants, 37 of 1010 (3.6%) with 1 variant, and 45 of 1277 (3.5%) with no variants had experienced AKI by the end of follow up.

Two *APOL1* variants (adjusted HR 2.97 [95% CI: 1.61–5.47]) and AKI (adjusted HR 22.7 [95%CI: 10.6–48.9]) as well as HIV viral load and CD4 cell count were associated with incident kidney failure (Supplementary Table S7). As the association between AKI and kidney failure varied by *APOL1* status (*P*_interaction_ = 0.001), the analyses were stratified by *APOL1* status (Table 5). In individuals with 2 *APOL1* variants, AKI was associated with a relatively modest 6-fold increase in kidney failure risk (adjusted HR 6.16 [95% CI: 1.9–19.7]). By comparison, AKI had a much stronger association with kidney failure in those with 1 and 0 risk variants (adjusted HR 245 [33–1799] and 68 [25–186] respectively). The associations between CD4 cell count and HIV viral load level with kidney failure were most pronounced among individuals with 2 *APOL1* variants.

**Table 5:** Factors associated with incident kidney failure, stratified by *APOL1* genotype

	0 *APOL1* risk allele; *n* AKI = 8, *n* ESKD = 29						1 *APOL1* risk allele, *n* AKI = 3, *n* ESKD = 11						2 *APOL1* risk alleles, *n* AKI ever = 8, *n* ESKD = 29					
	Univariable			Multivariable			Univariable			Multivariable			Univariable			Multivariable		
	HR	95% CI	*P* value	HR	95% CI	*P* value	HR	95% CI	*P* value	HR	95% CI	*P* value	HR	95% CI	*P* value	HR	95% CI	*P* value
Age (per yr)	0.98	0.95 – 1.02	0.37	0.98	0.95 – 1.02	0.31	1.01	0.95 – 1.07	0.24	0.98	0.93 – 1.05	0.68	0.94	0.91 – 0.97	0.0001	0.97	0.93 – 1.01	0.14
Sex (male)	1.65	0.79 – 3.42	0.2	1.19	0.54 – 2.65	0.80	3.43	0.91 – 12.9	0.68	1.99	0.46 – 8.43	0.35	1.72	0.82 – 3.61	0.1	1.40	0.57 – 3.42	0.490
AKI ever	54.3	21.9 – 134	< 0.001	68.42	25.18 – 185.96	< 0.001	112	24.1 – 523	< 0.001	244.8	33.3 – 1799	< 0.001	19.7	8.2 – 47	< 0.001	6.16	1.92 – 19.7	0.002
HIV RNA (time-updated)																		
< 50	1			1			1			1			1			1		
≥ 50	2.34	1.11 – 4.94	0.03	1.80	0.83 – 3.93	0.22	2.01	0.58 – 6.95	0.14	2.46	0.61 – 9.91	0.21	5.34	2.28 – 12.5	<0.001	3.35	1.31 – 8.54	0.01
CD4 cell count (time-updated)																		
< 50	1			1			1			1			1			1		
50–200	0.22	0.02 – 2.38	0.21	0.14	0.01 – 1.58	0.11	0.41	0.07 – 2.44	0.33	0.37	0.02 – 7.80	0.53	0.45	0.08 – 2.51	0.36	0.03	0.004 – 3.10	0.26
200–349	0.27	0.03 – 2.33	0.23	0.24	0.03 – 2.11	0.20	0.54	0.12 – 2.51	0.43	0.75	0.08 – 9.44	0.90	0.15	0.03 – 0.72	0.02	0.08	0.008 – 0.78	0.03
≥ 350	0.11	0.01 – 0.82	0.03	0.11	0.01 – 0.82	0.03	0.15	0.04 – 0.64	0.01	0.10	0.01 – 1.07	0.06	0.02	0.005 – 0.11	<0.001	0.03	0.004 – 0.31	0.003

AKI, acute kidney injury; ESKD, end-stage kidney disease; HR, hazard ratio.

Associations between stage 2/3 AKI, demographic/immune-virological parameters, and kidney failure during follow up (> 3 months from cohort inception).

## Discussion

We presented data from a large, well characterized cohort of people of African ancestry with HIV, who were followed up with serial creatinine measurements over a median of 13 years. Participants with 2 *APOL1* variants initiated HIV care with lower eGFR than those with 1 or 0 variants; they more often experienced AKI at inception, had more severe AKI and proteinuria at this time, and had poorer kidney function after AKI resolution during follow up. Participants with 2 *APOL1* variants were more likely to experience kidney failure during follow up. AKI was strongly associated with progression to kidney failure in participants with 1 and 0 *APOL1* variants whereas immune-virological markers were more strongly associated with kidney failure in those with 2 *APOL1* variants.

Two previous studies have reported an increased odds of AKI among hospitalized patients with COVID-19 who had *APOL1* high-risk genotypes.^14^^,^^15^ Our findings extend these observations to people with HIV. The early episodes of AKI are likely to have occurred in a setting of severe illness and acute inflammation not dissimilar to acute COVID-19, with AKI arising from kidney hypoperfusion, nephrotoxic medications and, in those with *APOL1* high-risk genotypes, variant APOL1 protein production in the kidney.^18^ This underscores the importance of pre-emptive AKI prevention in high-risk populations and highlights the potential role of *APOL1* genotyping in identifying individuals at increased risk, including those with HIV viraemia or sepsis. However, during follow up when HIV infection was generally well controlled, the risk of AKI was similar across all *APOL1* risk groups as previously reported for people without HIV.^19^ Of note, time-updated immunodeficiency remained strongly associated with AKI, a relationship that has previously been reported for eGFR decline in African Americans with *APOL1* high-risk genotypes^12^^,^^13^ and people with HIV and *APOL1* high-risk genotypes from Burkina Faso, Senegal, and Cameroon with high baseline HIV viral loads.^20^ Altogether, these data suggest that, in the absence of ongoing viral replication and immunodeficiency, the proinflammatory environment that leads to *APOL1* upregulation and cytotoxicity is attenuated, thereby reducing the differential impact of *APOL1* genotype on AKI risk and supporting the hypothesis that *APOL1*-associated kidney injury is not the result of constitutive *APOL1* expression but instead requires an environmental or inflammatory stimulus to become pathogenic.^21^

The risk of kidney failure associated with *APOL1* high-risk genotypes has been well described in otherwise healthy African American populations. Reports from the Chronic Renal Insufficiency Cohort,^20^ the African American Study of Kidney Disease and Hypertension,^22^ Atherosclerosis Risk in Communities study,^19^ and the Coronary Artery Risk Development in Young Adults cohort (26) have consistently demonstrated an increased risk of kidney failure among individuals with *APOL1* high-risk genotypes. More recent studies from the African continent, as well as consensus statements from a KDIGO working group, further support these findings.^23^^,^^24^ AKI is a recognized risk factor for kidney failure although this has not been reported for people with *APOL1* high-risk genotypes. We observed significant effect modification of the association between AKI and kidney failure by *APOL1* genotype, whereby AKI conferred a greater relative increase in kidney failure risk in those with 0 or 1 *APOL1* variants. A plausible explanation is that individuals with 2 *APOL1* variants already have a high underlying susceptibility to kidney failure, so AKI adds proportionally less additional risk. In contrast, those with 1 or 0 variants have lower baseline risk, meaning that AKI represents a more substantial insult and therefore leads to a much larger increase in the likelihood of kidney failure. Alternatively, those with *APOL1* high-risk genotypes may experience more gradual decline in kidney function over time. As a result, AKI episodes may not have met the study definition, or the additional impact of AKI on remaining kidney function may be relatively small. These findings highlight the importance of both genetic susceptibility and acquired kidney injury in driving kidney failure risk in populations of African ancestry with HIV.

Increased *APOL1* gene expression in podocytes in response to inflammation is well described,^25^ but the cellular and molecular mechanisms that lead to podocyte cytotoxicity remain unclear. Proposed mechanisms include disruptions in autophagy, increased lysosomal membrane permeability, mitochondrial dysfunction, endoplasmic reticulum stress, activation of stress-responsive kinases, and induction of pyroptotic cell death. More recently, *APOL1*-mediated cation flux has been directly implicated in cell death, both *in vitro*^26^ and *in vivo*.^27^ Notably, pharmacological inhibition of the *APOL1* ion channel in a clinical trial led to improved proteinuria in individuals with *APOL1* high-risk genotypes and focal segmental glomerulosclerosis,^28^ supporting the hypothesis that cytotoxic cation channel activity is a key driver of *APOL1*-mediated cell injury.

Several studies have reported an increased risk of focal segmental glomerulosclerosis^23^^,^^29^ and HIV-associated nephropathy^29^ among individuals with a single *APOL1* risk variant. However, the presence of 2 *APOL1* variants appears substantially more deleterious than 1 variant, with reported odds ratios for HIV-associated nephropathy, focal segmental glomerulosclerosis and CKD ranging from 3–89, 3–17, and 2–13 respectively, among individuals with 2 variants.^14^^,^^30^, ^31^, ^32^, ^33^ compared with 3.8, 1.6, and 1.2–4.4, respectively, among those with 1 variant.^23^^,^^29^^,^^34^ A single *APOL1* risk variant was not associated with reduced eGFR, kidney failure, proteinuria or HIV-associated nephropathy in our cohort,^8^ and not with AKI in the current analyses. Similarly, the Jackson Heart Study reported no association between a single *APOL1* variant and CKD outcomes,^35^ suggesting heterogeneity in monoallelic effects that may depend on population characteristics, comorbidities, or environmental exposures.

The strengths of our study include the large sample size, broad geographic representation of participants, and a long follow up period with access to serial serum creatinine measurements that allows precise tracking of kidney function decline over time. A major limiting factor of our study is its retrospective design. We lacked information on clinical events that lead or contributed to AKI, and we did not adjust for specific antiretroviral therapies (e.g., tenofovir disoproxil fumarate, atazanavir, lopinavir) that may influence kidney outcomes. However, these agents are typically avoided in individuals with or at risk of CKD^36^ and previous UK data (a setting with universal ART access) suggest no association between these potentially nephrotoxic regimens and AKI when prescribing practices account for underlying kidney disease.^2^ Although AKI episodes were ascertained using available creatinine data, we would have missed episodes of AKI that did not result in clinic attendance or hospitalization. Data completeness was high aside from uPCR. The substantial missingness in uPCR likely reflects real-world clinical practice, where proteinuria measurement is ordered selectively in patients with signs of kidney disease, abnormal eGFR, and comorbidities like diabetes, or where use of specific ARTs regimens may cause kidney disease. Nevertheless, effect estimates were consistent in analyses excluding uPCR, indicating that the missingness did not materially influence our conclusions. Because of the relatively small number of kidney failure events in some subgroups, particularly among those with both *APOL1* high-risk genotypes and AKI, estimates of interaction effects may be imprecise and should be interpreted with caution. Similarly, the number of AKI episodes at cohort inception was small potentially affecting the precision of the prevalence estimates. We could not determine the timing of diagnosis for key kidney risk factors such as hypertension and diabetes, which may have introduced residual confounding in analyses of AKI. Competing-risk modelling for death was not undertaken because of survivor bias inherent to the retrospective design— participants were required to be alive at the time of recruitment and *APOL1* genotyping (2018–2020) to be included in the present analyses.

In conclusion, this study shows that individuals with poorly controlled HIV and *APOL1* high-risk genotypes are at increased risk of AKI, more severe AKI, and poorer recovery of kidney function following AKI. AKI identifies individuals at elevated risk of kidney failure, providing opportunities for personalization of kidney care. Among people with HIV and *APOL1* high-risk genotypes, current approaches rely on continued suppression of HIV replication, avoiding ART medications with nephrotoxic potential, and nonspecific treatments to reduce proteinuria. As targeted *APOL1*-modifying therapies progress through development, they may in time enable more precise interventions to prevent acute and chronic *APOL1*-mediated cytotoxicity in the kidney.

## Appendix

### List of members of the GEN-AFRICA study group

**Barts Health NHS Trust, London** (John Booth [PI], Anele Waters, James Hand, Chris Clarke, Sarah Murphy, Maurice Murphy); **Brighton and Sussex University Hospitals, Brighton** (Marion Campbell, Amanda Clarke [PI], Celia Richardson, Alyson Knott, Gemma Weir, Rebecca Cleig, Helena Soviarova, Lisa Barbour, Tanya Adams, Vicky Kennard, Vittorio Trevitt); **Chelsea and Westminster Hospital, London** (Rachael Jones [PI], Alexandra Schoolmeester, Serah Duro; **Guy’s and St Thomas’ Hospital, London** (Rachel Hilton [PI], Julie Fox, May Rabuya, Lisa Hamzah, Deborah Jordan, Teresa Solano, Hiromi Uzu, Karen Williams, Julianne Lwanga, Linda Ekaette Reid-Amoruso, Hannah Gamlen, Robert J. Stocker, Fiona Ryan, Anele Waters, Karina Mahiouz, Tess Cheetham, Claire Williams, Achyuta Nori, Caroline Thomas, Sivaraj Venkateshwaran, Jessica Doctor, Andrea Berlanga); **King’s College Hospital, London** (Frank Post [CI], Beatriz Santana-Suarez, Leigh McQueen, Priya Bhagwandin, Lucy Campbell, Bee Barbini, Emily Wandolo, Tim Appleby, Deborah Jordan, Lois Driver, Sophy Parr, Hongbo Deng, Julie Barber, Andrew Crowe, Chris Taylor, Mary Poulton, Vida Boateng, Marie-Pierre Klein, Caitlin O'Brien, Samuel Ohene-Adomako, Christian Buckingham, Daniel Trotman, Killian Quinn, Kate Flanagan, Verity Sullivan, Holly Middleditch, Itty Samuel, Elizabeth Hamlyn, Candice McDonald, Ana Canoso, Emeka Agbasi, Maria Liskova, Sarah Barber, Amanda Samarawickrama, Zoe Ottaway, Claire Norcross, Amelia Oliveira, Kate Bramham); **Leeds Teaching Hospitals NHS Trust, Leeds** (Sarah Schoeman [PI], Gary Lamont, Ruby Cross, Gaushiya Saiyad, Shadia Ahmed, Rebecca Ashworth, Nicola Window, J Murira, Khine Phyu); **North Manchester General Hospital, Manchester** (Andrew Ustianowski [PI], Gabriella Lindergard, Jonathan Shaw, Sarah Holland, Claire Fox, Jan Flaherty, Margaret-Anne Bevan, Valerie George); **South Tees Hospitals NHS Foundation Trust, Middlesbrough** (David Chadwick [PI], Marie Branch, Pauline Lambert, Adele Craggs); **Mortimer Market Centre, Central and NorthWest London NHS Foundation Trust, London** (Sarah Pett [PI], Hinal Lukha, Nina Vora, Marzia Fiorino, Maria Muller Nunez, Deirdre Sally, James E. Burns, Erica Pool, Rebecca Matthews); **Newcastle upon Tyne Hospitals, Newcastle** (David Ashley Price [PI], Tara Stothard, Bijal Patel, Ian McVittie, Ciara Kennedy, Uli Shwab, Brendan Payne, Sarah Duncan, Jill Dixon, Mathias Schmid, Adam Evans, Christopher Duncan, Ewan Hunter, Yusri Taha, Natasha Astill); **National Cancer Institute, Frederick, USA** (Cheryl Winkler, Elizabeth Binns- Roemer, Victor David); **North Middlesex University Hospital, London** (Jonathan Ainsworth, Rachel Vincent [PI]); **Queen Elizabeth Hospital, Woolwich** (Stephen Kegg [PI], Chloe Saad, Sarah Skinner, Hocine Azzoug, Judith Russell, Tarik Moussaoui, Celia Richardson, Emily Mabonga, Donna Ward, J. Francoise, W. Larbi, Sue Mitchell, A. Manning, V. Russell); **Royal Free London Hospital, London** (Fiona Burns [PI], Mark Harber, Nnenna Ngwu, Jonathan Edwards, Nargis Hemat, Tom Fernandez, Filippo Ferro, Jorge Ferreira, Alice Nightingale, Tasha Oakes-Monger, Darwin Matila, Pedro Nogueira, Victoria Mutagwanya); **St. Georges University Hospitals, London** (Catherine Cosgrove [PI], Lisa Hamzah, Catherine Emily Isitt, Helen Webb, Joyce Popoola, Kate Korley, Mark Mencias, Patricia Ribeiro, Rajeshwar Ramkhelawn, Sandra Oliva Lara, Sara Sajijad); **Imperial College Healthcare NHS Trust, London** (Alan Winston [PI], Amber Shaw, Claire Petersen, Kyle Ring); **University Hospital Lewisham, London** (Melanie Rosenvinge [PI], Chloe Saad, Sarah Skinner, Thembi Moyo, Faith Odong, Katherine Gantert, Tina Ibe); and **Africa Advocacy Foundation** (Denis Onyango); **UK CHIC cohort** (Caroline Sabin [PI], Teresa Hill, Hajra Okhai).

## Disclosure

All the authors declared no competing interests.

## Supplementary Material

Supplementary file (PDF) **Figure S1.** Flow diagram of the participant numbers in the cohort. **Figure S2.** Kaplan-Meier curves showing the cumulative incidence of kidney failure: (A) overall cohort and (B) excluding prevalent kidney failure cases. **Table S1.** Characteristics of the study participants at cohort inception, stratified by AKI status at any time during follow-up. **Table S2.** Factors associated with AKI Stage 2/3 by *APOL1* status at cohort inception (full models). **Table S3.** Factors associated with AKI Stage 3 by *APOL1* status at cohort inception (full models). **Table S4.** Factors associated with AKI Stage 2/3 by *APOL1* status during follow up (full models). **Table S5.** Factors associated with AKI Stage 3 by *APOL1* status during follow up (full models). **Table S6.** Factors associated with AKI Stage 2/3 by *APOL1* status during follow up without urine PCR. **Table S7.** Factors associated with incident kidney failure. STROBE Checklist.

## Footnotes

Supplementary file (PDF)
**Figure S1.** Flow diagram of the participant numbers in the cohort. **Figure S2.** Kaplan-Meier curves showing the cumulative incidence of kidney failure: (A) overall cohort and (B) excluding prevalent kidney failure cases. **Table S1.** Characteristics of the study participants at cohort inception, stratified by AKI status at any time during follow-up. **Table S2.** Factors associated with AKI Stage 2/3 by *APOL1* status at cohort inception (full models). **Table S3.** Factors associated with AKI Stage 3 by *APOL1* status at cohort inception (full models). **Table S4.** Factors associated with AKI Stage 2/3 by *APOL1* status during follow up (full models). **Table S5.** Factors associated with AKI Stage 3 by *APOL1* status during follow up (full models). **Table S6.** Factors associated with AKI Stage 2/3 by *APOL1* status during follow up without urine PCR. **Table S7.** Factors associated with incident kidney failure. STROBE Checklist.
