# Supplementary material for: APOL1 Variants, AKI, and Progression to Kidney Failure in People of African Ancestry Living with HIV
Source: Kidney Int Rep. 2026 May 12;11(7):106580. doi: 10.1016/j.ekir.2026.106580 (PMC13314792; doi:10.1016/j.ekir.2026.106580)
Supplement: Supplementary file (PDF) — Figure S1. Flow diagram of the participant numbers in the cohort. Figure S2. Kaplan-Meier curves showing the cumulative incidence of kidney failure: (A) overall cohort and (B) excluding prevalent kidney failure cases. Table S1. Characteristics of the study participants at cohort inception, stratified by AKI status at any time during follow-up. Table S2. Factors associated with AKI Stage 2/3 by APOL1 status at cohort inception (full models). Table S3. Factors associated with AKI Stage 3 by APOL1 status at cohort inception (full models). Table S4. Factors associated with AKI Stage 2/3 by APOL1 status during follow up (full models). Table S5. Factors associated with AKI Stage 3 by APOL1 status during follow up (full models). Table S6. Factors associated with AKI Stage 2/3 by APOL1 status during follow up without urine PCR. Table S7. Factors associated with incident kidney failure. STROBE Checklist. [file mmc1.pdf]

APOL1 **Variants, Acute Kidney Injury, and Progression to Kidney Failure in People of African Ancestry with HIV**

Rachel K.Y. Hung MSc[1], John W. Booth PhD[2], Rachel Hilton FRCP[3], Lucy Campbell MSc[1], Prof. Julie Fox MD[1,3]; Andrew Ustianowski[4], Amanda Clarke FRCP[5,6], Lisa Hamzah PhD[7], Sarah Schoeman[8], Kate Bramham PhD[1,9], Prof. Fiona Burns[10], Hajra Okhai[10], Prof. Caroline A. Sabin PhD[10], Cheryl A. Winkler PhD[11] and Prof. Frank A. Post PhD[1,15] on behalf of the GEN-AFRICA study group

Affiliations

[1] King's College London, London, UK; [2] Barts Health NHS Trust, London, UK;[3]Guy's and St Thomas' NHS Foundation Trust, London, UK; [4]Pennine Acute Hospitals NHS Foundation Trust, Manchester; [5]Brighton and Sussex University Hospital NHS Trust, Brighton, UK; [6]Brighton and Sussex Medical School Department of Infectious Disease, Brighton, UK; [7]St George's Hospital NHS Foundation Trust, London, UK; [8]Leeds Teaching Hospitals NHS Trust, Leeds; [9]King's College Hospital NHS Foundation Trust, London, UK; [10]University College London, London, UK; [11] Cancer Innovation Laboratory, Center for Cancer Research and the Frederick National Laboratory for Cancer Research, National Cancer Institute, Frederick, MD, USA

Word count: 3889, 5 Tables (+4 suppl. Tables + 2 suppl. Figure)

Corresponding author

Dr Rachel Hung MBChB, MA, MSc, MRCP

King's College Hospital NHS Foundation Trust

Weston Education Centre (Rm 2.50), Cutcombe Road, London SE5 9RJ, United Kingdom

Email: rachel.hung@kcl.ac.uk

Table of Contents

Supplementary Table S1: Characteristics of the study participants at cohort inception, stratified by AKI status at any time during follow-up 3

Supplementary Table S2: Factors associated with AKI Stage 2/3 by *APOL1* status at cohort inception (full models) 4

Supplementary Table S3: Factors associated with AKI Stage 3 by *APOL1* status at cohort inception (full models) 5

Supplementary Table S4: Factors associated with AKI Stage 2/3 by *APOL1* status during follow up (full models) 6

Supplementary Table S5: Factors associated with AKI Stage 3 by *APOL1* status during follow up (full models) 7

Supplementary Table S6: Factors associated with AKI Stage 2/3 by *APOL1* status during follow up without urine PCR 8

Supplementary Table S7: Factors associated with incident kidney failure 9

Supplementary Figure S1: Flow diagram of the participant numbers in the cohort 10

Supplementary Figure S2: Kaplan-Meier curves showing the cumulative incidence of kidney failure: (a) overall cohort and (b) excluding prevalent kidney failure cases 11

Supplementary Table S1: Characteristics of the study participants at cohort inception, stratified by AKI status at any time during follow-up

		All (N=2701)	No AKI (N=2536)	AKI [a] (N=165)	*P*-value
***APOL1* status**					<0.001
0 variant	n (%)	1325 (49.1)	1258 (49.6)	67 (40.6)	
1 variant	n (%)	1033 (38.2)	976 (38.5)	57 (34.5)	
2 variants	n (%)	343 (12.7)	302 (11.9)	41 (24.8)	
**Age**	Mean (SD)	38.7 (10.8)	38.6 (10.8)	39.9 (10.5)	0.1
**Sex (Male)**	n (%)	1168 (43.2)	1083 (42.7)	85 (51.5)	0.03
**At cohort inception**					
**HIV Status**					0.07
New HIV Diagnosis	n (%)	1467 (54.3)	1370 (54.0)	97 (58.8)	
Reengagement with care	n (%)	233 (8.6)	211 (8.3)	22 (13.3)	
Transfer into Care	n (%)	999 (37.0)	953 (37.6)	46 (27.9)	
**CD4 cell count (cells/mm3)**	Median (IQR)	307 [122 – 509]	313 [135 – 513]	120 [38.9 – 388]	<0.001
**HIV viral load (log copies/mL)**	Median (IQR)	3.7 [1.7 – 4.8]	3.69 [1.7 – 4.8]	4.1 [2.1 – 5.4]	<0.001
**eGFR (mL/min/1.73m3)**	Median (IQR)	95 [78 – 110]	95 [79 – 110]	72 [29 – 101]	<0.001
**uPCR (mg/mmol)**	Median (IQR)	11 [7 – 23]	10 [7 – 20]	69 [17 – 320]	<0.001

eGFR=estimated glomerular filtration rate (CKD-EPI-2021)

[a] This group comprises participants who experienced one or more episodes of AKI at any time from cohort inception

NB: HIV viral load is expressed as log copies/mL (a viral load of 50 equates to log 1.7)

Supplementary Table S2: Factors associated with AKI Stage 2/3 by *APOL1* status at cohort inception (full models)

AKI <3 months of inception	Univariable			Multivariable					
	PR	95% CI	*P* value	PR	95% CI	*P* value	PR	95% CI	*P* value
**1 *APOL1* variant**	1.36	069 - 2.68	0.37	1.35	0.68 - 2.68	0.38	1.25	0.55 - 2.82	0.59
**2 *APOL1* variants**	6.28	3.41 - 11.57	<0.001	6.2	3.35 - 11.49	<0.001	6.91	3.46 - 13.81	<0.001
**Sex (male)**	1.19	0.71 - 1.97	0.51	1.10	0.67 - 1.83	0.70	1.07	0.62 - 1.84	0.80
**Age (per year)**	1.01	0.98 - 1.03	0.47	1.01	0.98 - 1.03	0.61	1.10	0.64 - 1.88	0.72
**HIV viral load (copies/mL)**									
<5000	1						1		
5000-100,000	0.61	0.19 - 1.94	0.4				0.33	0.09 - 1.16	0.08
≥100,000	6.3	3.13 - 12.7	<0.001				2.57	1.07 - 6.08	0.03
**CD4 cell count (cells/mm^3)**									
<50	1						1		
50-200	0.41	0.22 - 0.76	<0.001				0.43	0.21 - 0.87	0.02
200-349	0.19	0.08 - 0.43	<0.001				0.32	0.11 - 0.91	0.03
≥350	0.1	0.05 - 0.23	<0.001				0.19	0.07 - 0.54	0.002

Associations between demographic and immune-virological parameters and stage 2/3 AKI at (within 3 months of) cohort inception.

PR=prevalence ratio.

Supplementary Table S3: Factors associated with AKI Stage 3 by *APOL1* status at cohort inception (full models)

**AKI3 <3 months of inception**	**Univariate**			**Multivariable**					
	**PR**	**95% CI**	***P* value**	**PR**	**95% CI**	***P* value**	**PR**	**95% CI**	***P* value**
**1 *APOL1* variant**	1.49	0.69 - 3.22	0.3	1.49	0.69 - 3.23	0.31	1.29	0.50 - 3.35	0.60
**2 *APOL1* variants**	7.40	3.72 - 14.73	<0.001	7.33	3.65 - 14.73	<0.001	9.22	4.19 - 20.29	<0.001
**Sex (male)**	1.26	0.72 - 2.19	0.41	1.18	0.67 - 2.06	0.56	1.18	0.65 - 2.13	0.51
**Age (per year)**	1.00	0.98 - 1.03	0.71	1	0.99 - 1.02	0.98	0.99	0.97 - 1.03	0.95
**HIV viral load (copies/mL)**									
<5000	1						1		
5000-100,000	0.57	0.15 - 2.15	0.41				0.25	0.05 - 1.17	0.07
≥100,000	6.92	3.19 - 15.03	<0.001				2.66	0.98 - 7.22	0.05
**CD4 cell count (cells/mm^3)**									
<50	1						1		
50-200	0.41	0.20 - 0.81	0.001				0.42	0.19 - 0.89	0.02
200-349	0.23	0.009 - 0.53	<0.001				0.39	0.14 - 1.16	0.09
≥350	0.09	0.03 - 0.24	<0.001				0.15	0.04 - 0.55	0.004

Associations between demographic and immune-virological parameters and stage 3 AKI at (within 3 months of) cohort inception.

PR=prevalence ratio.

Supplementary Table S4: Factors associated with AKI Stage 2/3 by *APOL1* status during follow up (full models)

AK 2/3 >3 months from inception	Univariate			Demographics			+ HIV factors			+ Kidney factors		
	**IRR**	**95% CI**	***P* value**	**IRR**	**95% CI**	***P* value**	**IRR**	**95% CI**	***P* value**	**IRR**	**95% CI**	***P* value**
**1 *APOL1* variant**	1.21	0.83 - 1.77	0.33	1.17	0.80 - 1.70	0.43	1.11	0.75 - 1.63	0.61	0.82	0.37 - 1.84	0.63
**2 *APOL1* variants**	1.67	0.99 - 2.84	0.06	1.61	0.94 - 2.74	0.08	1.44	0.81 - 2.57	0.22	1.67	0.61 - 4.60	0.32
**Sex (male)**	1.67	1.18 - 2.38	0.004	1.62	1.14 - 2.31	0.007	1.41	0.97 - 2.05	0.07	0.68	0.32 - 1.47	0.33
**Age (per year)**	1.01	0.99 - 1.03	0.36	1.01	0.99 - 1.02	0.51	1.02	1.00 - 1.04	0.02	1.00	0.96 - 1.03	0.80
**HIV viral load (copies/mL; time-updated)**												
<50	1						1			1		
≥50	1.57	1.05 - 2.34	0.03				1.37	0.91 - 2.04	0.13	1.16	0.55 - 2.43	0.69
**CD4 cell count (cells/mm3; time-updated)**												
<50	1						1			1		
50-200	0.35	0.18 - 0.68	0.002				0.41	0.20 - 0.86	0.02	0.41	0.10 - 1.64	0.21
200-349	0.17	0.09 - 0.33	<0.001				0.20	0.10 - 0.41	<0.001	0.11	0.02 - 0.52	0.005
≥350	0.05	0.03 - 0.09	<0.001				0.07	0.03 - 0.13	<0.001	0.07	0.02 - 0.23	<0.001
**eGFR (mL/min/1.73m^2; time-updated)**												
≥90	1						1			1		
60-89	1.07	2.53 - 6.15	0.74				1.15	0.74 - 1.77	0.54	1.14	0.43 - 3.03	0.79
<60	3.94	2.553 - 6.15	<0.001				3.47	2.02 - 5.94	<0.001	3.30	1.05 - 10.39	0.04
**uPCR (mg/mmol; time-updated)**												
<15	1									1		
15-49	1.80	0.77 - 4.20	0.18							1.55	0.65 - 3.72	0.33
50-99	2.46	0.57 -10.68	0.23							1.54	0.31 - 7.80	0.60
≥100	8.7	3.72 - 20.32	<0.001							3.87	1.36 - 11.01	0.01

Associations between demographic, immune-virological and kidney function parameters and stage 2/3 AKI at during follow up (>3 months from cohort inception). IRR=incidence rate ratio.

Supplementary Table S5: Factors associated with AKI Stage 3 by *APOL1* status during follow up (full models)

AKI3 >3 months from inception	Univariate			Demographics			+ HIV factors			+ Kidney factors		
	IRR	95% CI	*P* value	IRR	95% CI	*P* value	IRR	95% CI	*P* value	IRR	95% CI	*P* value
**1 *APOL1* variant**	1.13	0.63 - 2.03	0.69	1.09	0.61 - 1.96	0.76	0.99	0.53 - 1.86	0.98	0.68	0.19 - 2.45	0.55
**2 *APOL1* variants**	2.54	1.28 - 5.06	0.008	2.47	1.24 - 4.91	0.01	2.58	1.25 - 5.32	0.01	1.33	0.30 - 5.83	0.71
**Sex (male)**	1.47	0.88 - 2.48	0.14	1.40	0.84 - 2.34	0.19	1.11	0.64 - 1.93	0.7	0.61	0.22 - 1.64	0.33
**Age (per year)**	1.01	0.98 - 1.047	0.38	1.01	0.98 - 1.04	0.46	1.03	1.01 - 1.06	0.02	0.97	0.94 - 1.00	0.09
**HIV viral load (copies/mL; time-updated)**												
<50	1						1			1		
≥50	1.72	0.98 - 3.18	0.08				1.65	0.89 - 3.05	0.11	0.87	0.32 - 2.35	0.78
**CD4 cell count (cells/mm3; time-updated)**												
<50	1						1			1		
50-200	0.31	0.12 - 0.80	0.02				0.53	0.14 - 1.97	0.34	0.79	0.09 - 7.28	0.84
200-349	0.14	0.06 - 0.37	<0.001				0.28	0.08 - 1.01	0.05	0.09	0.01- 1.06	0.10
≥350	0.05	0.02 - 0.12	<0.001				0.10	0.03 - 0.33	<0.001	0.11	0.01 - 0.89	0.04
**eGFR (mL/min/1.73m^2; time-updated)**												
≥90	1									1		
60-89	1.00	0.50 - 2.00	0.99							0.88	0.14 - 5.29	0.89
<60	8.35	4.57 - 15.24	<0.001							7.18	1.59 - 32.43	0.01
**uPCR (mg/mmol; time-updated)**												
<15	1									1		
15-49	1.2	0.24 - 5.94	0.82							1.02	0.21 - 4.96	0.98
50-99	6.55	1.32 - 32.45	0.02							3.40	0.51 - 22.61	0.21
≥100	17.39	5.60 - 54.05	<0.001							5.28	1.44 - 19.41	0.01

Associations between demographic, immune-virological and kidney function parameters and stage 3 AKI at during follow up (>3 months from cohort inception). IRR=incidence rate ratio.

Supplementary Table S6: Factors associated with AKI Stage 2/3 by *APOL1* status during follow up without urine PCR

AKI >3 months from inception	Univariate			Demographics			+ HIV factors		
	**IRR**	**95% CI**	***P* value**	**IRR**	**95% CI**	***P* value**	**IRR**	**95% CI**	***P* value**
**1 *APOL1* variant**	1.21	0.83 - 1.77	0.33	1.17	0.80 - 1.70	0.43	1.03	0.69 - 1.52	0.89
**2 *APOL1* variants**	1.67	0.99 - 2.84	0.06	1.61	0.94 - 2.74	0.08	1.24	0.70 - 2.20	0.46
**Sex (male)**	1.67	1.18 - 2.38	0.004	1.62	1.14 - 2.31	0.007	1.44	0.99 - 2.09	0.06
**Age (per year)**	1.01	0.99 - 1.03	0.36	1.01	0.99 - 1.02	0.51	1.01	1.00 - 1.03	0.12
**HIV RNA**									
<50	1						1		
50- 199	0.95	0.60 - 1.50	0.83				1.01	0.64 - 1.60	0.98
200-999	1.49	0.58 - 3.80	0.40				1.30	0.49 - 3.47	0.60
1,00-99,999	2.98	1.70 - 5.21	<0.001				2.61	1.42 - 4.81	0.002
≥100,000	13.15	7.5 - 23.06	<0.001				7.43	3.55 - 15.55	<0.001
**CD4 cell count**									
<50	1						1		
50-200	0.35	0.18 - 0.68	0.002				0.71	0.32 - 1.57	0.39
200-349	0.17	0.09 - 0.33	<0.001				0.48	0.20 - 1.13	0.09
≥350	0.05	0.03 - 0.09	<0.001				0.20	0.09 - 0.45	<0.001
**eGFR (time-updated)**									
≥90	1						1		
60-90	1.07	2.53 - 6.15	0.74				1.15	0.74 - 1.77	0.54
<60	3.94	2.553 - 6.15	<0.001				3.47	2.02 - 5.94	<0.001

Associations between demographic, immune-virological and kidney function parameters and stage 2/3 AKI at during follow up but not adjusted for urine PCR (>3 months from cohort inception). IRR=incidence rate ratio.

Supplementary Table S7: Factors associated with incident kidney failure

	Univariable			Multivariable		
	**HR**	**95% CI**	***P* value**	**HR**	**95% CI**	***P* value**
**Age (per year)**	0.97	0.95 - 0.99	0.008	0.99	0.96 - 1.01	0.43
**Sex (male)**	1.92	1.19 - 3.09	0.007	1.52	0.89 - 2.57	0.37
**1 *APOL1* variant**	0.50	0.25 - 1.00	0.05	0.49	0.23 - 1.03	0.05
**2 *APOL1* variants**	4.79	2.86 - 8.03	<0.001	2.97	1.61 - 5.47	<0.001
**AKI ever**	58.5	32.9 - 104	<0.001	22.7	10.6 - 48.9	<0.001
**HIV RNA (time-updated)**						
<50	1			1		
50- 199	2.25	1.26 - 4.01	0.006	2.04	1.14 - 3.65	0.02
200-999	0.87	0.12 = 6.43	0.89	0.73	0.09 - 5.51	0.75
1,00-99,999	5.88	2.75 - 12.55	<0.001	2.14	0.89 - 6.84	0.08
≥100,000	15.2	5.83 - 39.58	<0.001	1.83	0.69 - 4.84	0.22
**CD4 cell count (time-updated)**						
<50	1			1		
50-200	0.46	0.13 - 1.57	0.22	0.27	0.06 - 1.15	0.10
200-349	0.46	0.15 - 1.67	0.16	0.19	0.05 - 0.75	0.02
≥350	0.10	0.04 - 0.29	<0.001	0.09	0.03 - 0.35	<0.001

HR=hazard ratio. The HR for participants with 1 and 2 *APOL1* variants are expressed relative to those with 0 variants. **The association between AKI and kidney failure varied by *APOL1* status ($P_{interaction}$ = 0.001).**

Supplementary Figure S1: Flow diagram of the participant numbers in the cohort

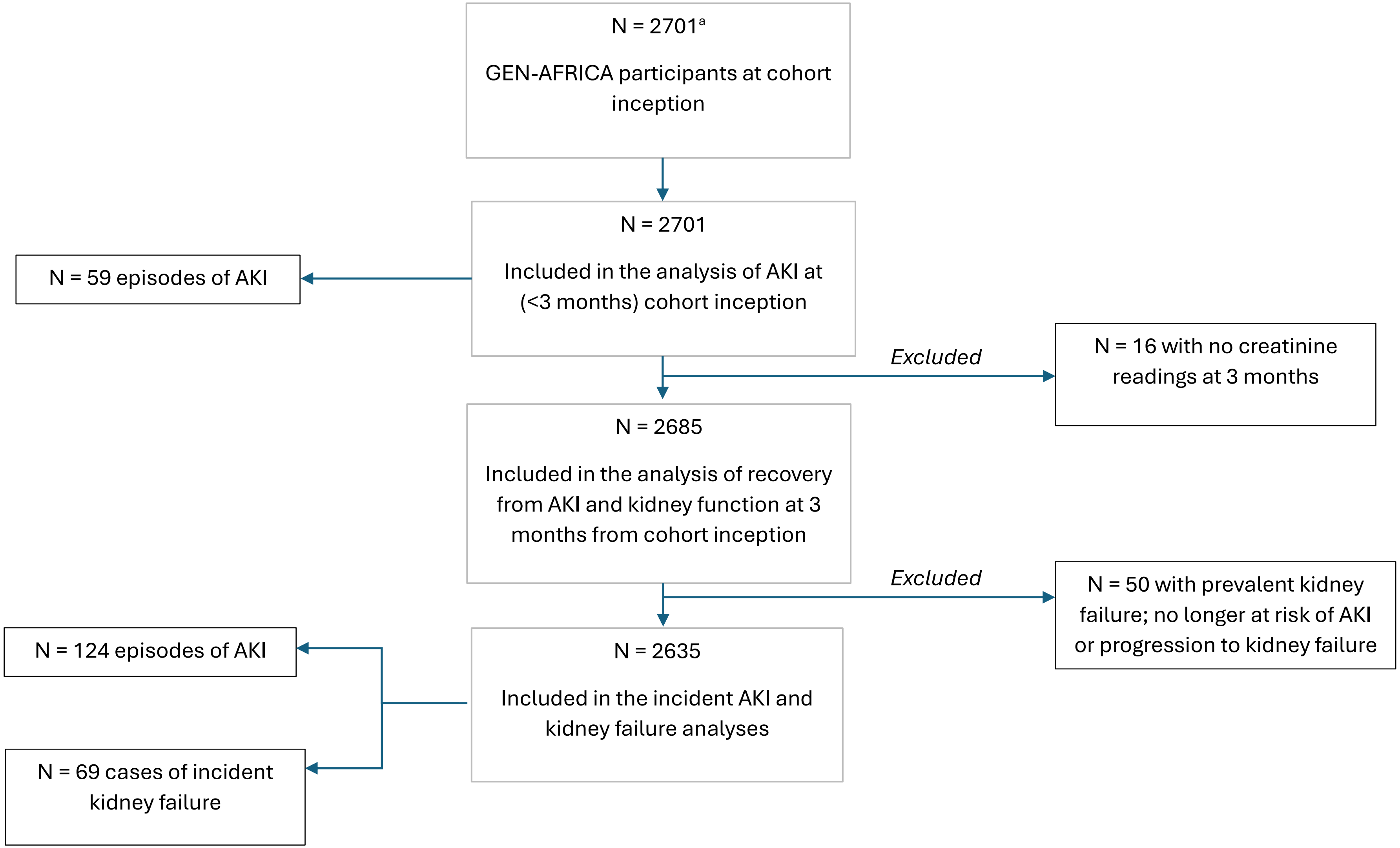

[a]A total of 3027 individuals were enrolled in the GEN-AFRICA study; *APOL1* genotyping was successful for 2864 (94.6%). However, only 2,701 individuals had creatinine readings available prior to enrolment into the GEN AFRICA study

Supplementary Figure S2: Kaplan-Meier curves showing the cumulative incidence of kidney failure: (a) overall cohort and (b) excluding prevalent kidney failure cases

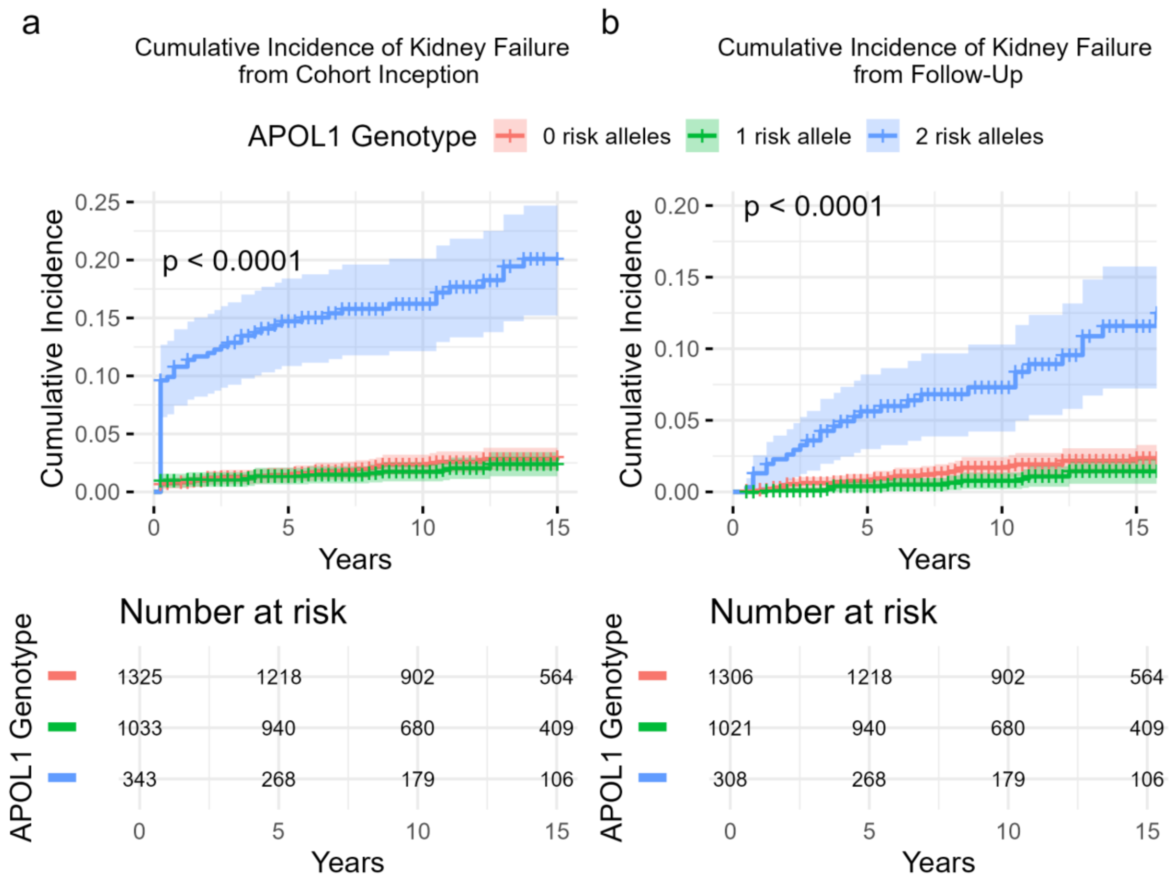

STROBE Statement—Checklist of items that should be included in reports of ***cohort studies***

	Item No	Recommendation	Page No
**Title and abstract**	1	(*a*) Indicate the study's design with a commonly used term in the title or the abstract	2
		(*b*) Provide in the abstract an informative and balanced summary of what was done and what was found	2
**Introduction**			
Background/rationale	2	Explain the scientific background and rationale for the investigation being reported	3
Objectives	3	State specific objectives, including any prespecified hypotheses	3
**Methods**			
Study design	4	Present key elements of study design early in the paper	3-5
Setting	5	Describe the setting, locations, and relevant dates, including periods of recruitment, exposure, follow-up, and data collection	3-5
Participants	6	(*a*) Give the eligibility criteria, and the sources and methods of selection of participants. Describe methods of follow-up	3-5
		(*b*) For matched studies, give matching criteria and number of exposed and unexposed	
Variables	7	Clearly define all outcomes, exposures, predictors, potential confounders, and effect modifiers. Give diagnostic criteria, if applicable	3-5
Data sources/ measurement	8*	For each variable of interest, give sources of data and details of methods of assessment (measurement). Describe comparability of assessment methods if there is more than one group	3-5
Bias	9	Describe any efforts to address potential sources of bias	3-5
Study size	10	Explain how the study size was arrived at	3-5
Quantitative variables	11	Explain how quantitative variables were handled in the analyses. If applicable, describe which groupings were chosen and why	3-5
Statistical methods	12	(*a*) Describe all statistical methods, including those used to control for confounding	3-5
		(*b*) Describe any methods used to examine subgroups and interactions	
		(*c*) Explain how missing data were addressed	
		(*d*) If applicable, explain how loss to follow-up was addressed	
		(*e*) Describe any sensitivity analyses	
**Results**			

Participants	13*	(a) Report numbers of individuals at each stage of study—eg numbers potentially eligible, examined for eligibility, confirmed eligible, included in the study, completing follow-up, and analysed<br><br>(b) Give reasons for non-participation at each stage<br><br>(c) Consider use of a flow diagram	6-8
Descriptive data	14*	(a) Give characteristics of study participants (eg demographic, clinical, social) and information on exposures and potential confounders<br><br>(b) Indicate number of participants with missing data for each variable of interest<br><br>(c) Summarise follow-up time (eg, average and total amount)	6-8
Outcome data	15*	Report numbers of outcome events or summary measures over time	6-8

Main results	16	(*a*) Give unadjusted estimates and, if applicable, confounder-adjusted estimates and their precision (eg, 95% confidence interval). Make clear which confounders were adjusted for and why they were included<br>(*b*) Report category boundaries when continuous variables were categorized<br>(*c*) If relevant, consider translating estimates of relative risk into absolute risk for a meaningful time period	6-8
Other analyses	17	Report other analyses done—eg analyses of subgroups and interactions, and sensitivity analyses	6-8
**Discussion**			
Key results	18	Summarise key results with reference to study objectives	9-10
Limitations	19	Discuss limitations of the study, taking into account sources of potential bias or imprecision. Discuss both direction and magnitude of any potential bias	10-11
Interpretation	20	Give a cautious overall interpretation of results considering objectives, limitations, multiplicity of analyses, results from similar studies, and other relevant evidence	9-11
Generalisability	21	Discuss the generalisability (external validity) of the study results	10-11
**Other information**			
Funding	22	Give the source of funding and the role of the funders for the present study and, if applicable, for the original study on which the present article is based	12

*Give information separately for exposed and unexposed groups.

Note: An Explanation and Elaboration article discusses each checklist item and gives methodological background and published examples of transparent reporting. The STROBE checklist is best used in conjunction with this article (freely available on the Web sites of PLoS Medicine at http://www.plosmedicine.org/, Annals of Internal Medicine at http://www.annals.org/, and Epidemiology at http://www.epidem.com/). Information on the STROBE Initiative is available at http://www.strobe-statement.org.
